# Contributions of gut bacteria to *Bacillus thuringiensis*-induced mortality vary across a range of Lepidoptera

**DOI:** 10.1186/1741-7007-7-11

**Published:** 2009-03-04

**Authors:** Nichole A Broderick, Courtney J Robinson, Matthew D McMahon, Jonathan Holt, Jo Handelsman, Kenneth F Raffa

**Affiliations:** 1 Department of Entomology, University of Wisconsin, Madison, WI 53706, USA; 2 Microbiology Doctoral Training Program, University of Wisconsin, Madison, WI 53706, USA; 3 Department of Bacteriology, University of Wisconsin, Madison, WI 53706, USA

## Abstract

**Background:**

Gut microbiota contribute to the health of their hosts, and alterations in the composition of this microbiota can lead to disease. Previously, we demonstrated that indigenous gut bacteria were required for the insecticidal toxin of *Bacillus thuringiensis *to kill the gypsy moth, *Lymantria dispar*. *B. thuringiensis *and its associated insecticidal toxins are commonly used for the control of lepidopteran pests. A variety of factors associated with the insect host, *B. thuringiensis *strain, and environment affect the wide range of susceptibilities among Lepidoptera, but the interaction of gut bacteria with these factors is not understood. To assess the contribution of gut bacteria to *B. thuringiensis *susceptibility across a range of Lepidoptera we examined larval mortality of six species in the presence and absence of their indigenous gut bacteria. We then assessed the effect of feeding an enteric bacterium isolated from *L. dispar *on larval mortality following ingestion of *B. thuringiensis *toxin.

**Results:**

Oral administration of antibiotics reduced larval mortality due to *B. thuringiensis *in five of six species tested. These included *Vanessa cardui *(L.)*, Manduca sexta *(L.)*, Pieris rapae *(L.) and *Heliothis virescens *(F.) treated with a formulation composed of *B. thuringiensis *cells and toxins (DiPel), and *Lymantria dispar *(L.) treated with a cell-free formulation of *B. thuringiensis *toxin (MVPII). Antibiotics eliminated populations of gut bacteria below detectable levels in each of the insects, with the exception of *H. virescens*, which did not have detectable gut bacteria prior to treatment. Oral administration of the Gram-negative *Enterobacter sp*. NAB3, an indigenous gut resident of *L. dispar*, restored larval mortality in all four of the species in which antibiotics both reduced susceptibility to *B. thuringiensis *and eliminated gut bacteria, but not in *H. virescens*. In contrast, ingestion of *B. thuringiensis *toxin (MVPII) following antibiotic treatment significantly increased mortality of *Pectinophora gossypiella *(Saunders), which was also the only species with detectable gut bacteria that lacked a Gram-negative component. Further, mortality of *P. gossypiella *larvae reared on diet amended with *B. thuringiensis *toxin and *Enterobacter sp*. NAB3 was generally faster than with *B. thuringiensis *toxin alone.

**Conclusion:**

This study demonstrates that in some larval species, indigenous gut bacteria contribute to *B. thuringiensis *susceptibility. Moreover, the contribution of enteric bacteria to host mortality suggests that perturbations caused by toxin feeding induce otherwise benign gut bacteria to exert pathogenic effects. The interaction between *B. thuringiensis *and the gut microbiota of Lepidoptera may provide a useful model with which to identify the factors involved in such transitions.

## Background

Since the independent discovery of *Bacillus thuringiensis *in two lepidopteran species, *Bombyx mori *(L.) and *Ephestia kuehniella *(Zeller), at the beginning of the 20^th ^century [1,2], Lepidoptera have served as the leading insect model for elucidating the mode of action and specificity of *B. thuringiensis *and its associated insecticidal toxins. A variety of lepidopteran whole-animal models, as well as cell lines and membrane preparations derived from Lepidoptera, have been used to identify factors that enhance or inhibit *B. thuringiensis *activity and define the cellular and molecular responses to *B. thuringiensis *toxin. In particular, the use of lepidopteran cell culture and brush border membrane vesicle preparations to dissect the complex interactions between toxin and midgut receptors have generated a substantive understanding of the mechanisms of pore formation and defined the processes that lead to disruption of larval gut integrity [3-5]. In combination with studies on resistant insects, studies in these models have also identified the specific midgut receptors involved in toxin binding, including cadherin-like proteins, aminopeptidases, and additional GPI-anchored proteins such as alkaline phosphatase [6-11]. These studies have also demonstrated that susceptibility to *B. thuringiensis *varies among different species of Lepidoptera, in the amount of toxin required to cause mortality, the speed of mortality, and the response to toxin following ingestion [12,13].

Beginning with their original characterization, Heimpel and Angus described *B. thuringiensis *as exhibiting variable modes of action, and thus categorized lepidopteran host species into three groups [14]. Type I species exhibit a general paralysis following ingestion of toxin and rapid death within hours. Type II species, which include the majority of Lepidoptera, are characterized by a cessation of feeding following toxin ingestion, a paralysis restricted to the gut, and death within 2 to 4 days. Type III insects require both spore and toxin for mortality by *B. thuringiensis*. Subsequent analyses demonstrated additional complexity in host responses, as factors such as toxin concentration and larval age can result in variable modes of action even within the same insect species (for example, toxic action at high doses, septicemia at low doses) [15-17]. In addition, certain physiological and genetic features of lepidopteran hosts are known to contribute to differences in susceptibility to *B. thuringiensis*. For example, host factors such as midgut pH and proteases contribute to the solubilization and activation of toxin following ingestion.

The beneficial contributions of gut microbiota to host health are generally acknowledged [18-21]. However, they can also have negative impacts, as perturbations in the composition or location of gut microbiota can lead to pathological states and host mortality [22-26]. Previously, we demonstrated that the elimination of indigenous enteric bacteria from gypsy moth larvae, *Lymantria dispar *(L.), achieved by rearing them on antibiotics, greatly reduced susceptibility to a formulation of *B. thuringiensis *(DiPel) composed of cells, spores, and toxins (Cry1Aa, Cry1Ab, Cry1Ac, and Cry2A) [27]. Additionally, re-establishment of a single enteric species, *Enterobacter *sp. NAB3, restored *B. thuringiensis *susceptibility. To account for a potential direct impact of antibiotics on the *B. thuringiensis *bacterium, we used an *Escherichia coli *strain engineered to produce the *B. thuringiensis *Cry1Aa toxin gene. Ingestion of an overnight culture containing live, but not heat-killed, *E. coli *Cry1Aa-producing cells also restored susceptibility of larvae reared on antibiotics to *B. thuringiensis*. Additionally, prior rearing on antibiotics did not inhibit establishment of *Enterobacter *sp. NAB3 when fed to larvae after cessation of antibiotic feeding, even though this strain is sensitive to the antibiotics incorporated into artificial diet [28], suggesting that direct effects of antibiotics on *B. thuringiensis *cells during the feedback are also unlikely. This interpretation is supported by our direct measurements (not reported) of an average of 2 × 10^2 ^CFU/gut of *B. thuringiensis *from larvae fed antibiotics, compared with 1.6 × 10^2 ^CFU/gut in larvae reared without antibiotics. Based on this evidence and our distinction between how *B. thuringiensis *was unable to grow in the hemolymph of living larvae even though it can grow rapidly in dead or moribund larvae [12,27,29-35], we proposed a model in which toxin disruption of the midgut leads to septicemia by enteric bacteria resulting in both larval death and more favorable conditions for *B. thuringiensis *germination and growth. The broader applicability of this model to additional lepidopteran species was unknown.

To determine whether enteric bacteria were necessary for susceptibility to *B. thuringiensis *in other Lepidoptera, we compared larval mortality to *B. thuringiensis *in the presence and absence of their indigenous gut bacteria among a range of species from five additional families. These included: *Manduca sexta *(L.) (Sphingidae)*, Vanessa cardui *(L.) (Nymphalidae)*, Pieris rapae *(L.) (Pieridae)*, Heliothis virescens *(F.) (Noctuidae), and *Pectinophora gossypiella *(Saunders) (Gelechiidae). We also used a cell-free formulation of *B. thuringiensis*, MVPII, in assays with *L. dispar *to extend our work with the DiPel cell-based formulation. The MVPII formulation consists of Cry1Ac protoxin encapsulated in NaCl-killed *Pseudomonas fluorescens *cells. MVPII was also used in assays with *Pectinophora gossypiella*, as it is the most commonly used *B. thuringiensis *formulation with this species [36]. We then assessed the ability of a single enteric bacterium from *L. dispar*, *Enterobacter sp*. NAB3, to restore *B. thuringiensis*-induced killing in those species in which antibiotics reduced mortality. Additionally, we characterized the enteric bacteria associated with larvae of each species and determined the effects of our antibiotic treatments on these communities.

## Results

### Diversity of gut bacteria and effects of antibiotic treatments among test Lepidoptera

Phylogenetic analysis of 16S rRNA gene sequences obtained from clone libraries from the midguts of *V. cardui*, *M. sexta*, *P. rapae*, and *P. gossypiella *larvae indicated relatively narrow taxonomic diversity of gut bacteria (Table 1). Collectively, all 16S rRNA gene sequences affiliated with either Gram-positive Firmicutes, representing only two species (*Lactococcus lactis *and *Enterococcus casseliflavus*) or Gram-negative bacteria within the γ-Proteobacteria subphylum. Amongst the γ-Proteobacteria, all 16S rRNA gene sequences affiliated with the family Enterobacteriaceae except for one, which was affiliated with *Pseudomonas putida *(Pseudomonadaceae). In addition, the compositions of the gut communities of these Lepidoptera were relatively simple, generally consisting of two bacterial phylotypes, one of which was an Enterobacteriaceae, except in the case of *P. gossypiella*, which was singularly associated with the Gram-positive bacterium *E. casseliflavus*. No gut bacteria were identified in *H. virescens *guts either by culturing or direct 16S rRNA gene analysis. Rearing larvae on antibiotics reduced gut bacteria to below detectable levels in all five Lepidoptera species in which they were previously detectable (Table 1).

**Table 1:** Enteric bacteria in larvae of five lepidopteran species identified by 16S rRNA gene sequence analysis.

**Larval species**		**Bacterial species detected in guts of larvae reared on:**	
**Family**	**Species**	**sterile artificial diet**	**diet with antibiotics**

Nymphalidae	*Vanessa cardui*	*Lactococcus lactis*	none detected
		*Klebsiella *sp.	
Sphingidae	*Manduca sexta*	*Enterobacter *sp.	none detected
		*Klebsiella *sp.	
Pieridae	*Pieris rapae*	*Enterobacter *sp.	none detected
		*Pantoea *sp.	
Noctuidae	*Heliothis virescens*	none detected	none detected
Gelechiidae	*Pectinophora gossypiella*	*Enterococcus casseliflavus*	none detected
Lymantriidae	*Lymantria dispar*	*Enterobacter *sp. NAB3	none detected
		*Pseudomonas putida*	

### Effect of antibiotics on susceptibility of Lepidoptera to *B. thuringiensis*

**Figure 1:**
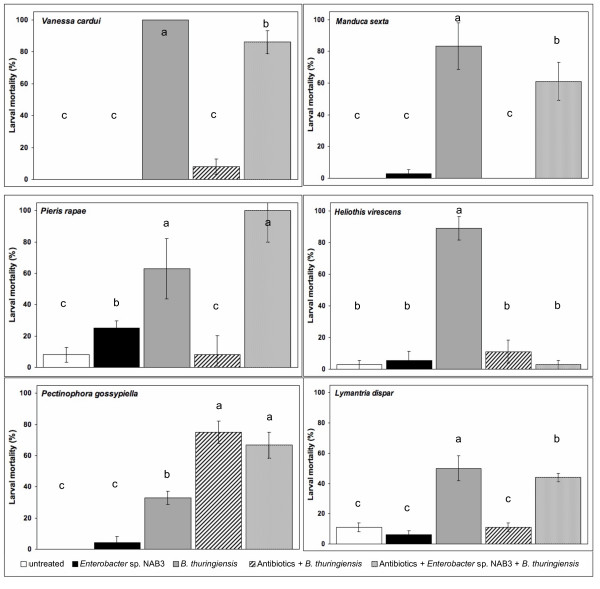
**Effect of antibiotics and *Enterobacter *sp. NAB3 on susceptibility of six Lepidoptera species to *Bacillus thuringiensis***. Mortality for larvae of each species was analyzed by ANOVA. Each bar represents the mean mortality ± SEM of 36 larvae (three replications with 12 larvae each). Means were separated for significance according to Fisher's protected LSD at *P *< 0.05 (*Vc*: *F *= 189.11, df = 4, *P *< 0.0001; *Ms*: F = 47.32, df = 4, *P *< 0.0001; *Pr*: *F *= 280.37, df = 4, *P *< 0.0001; *Hv*: *F *= 93.07, df = 4, *P *< 0.0001; *Pg*: *F *= 36.21, df = 4, *P *< 0.0001; *Ld*: *F *= 47.32, df = 4, *P *< 0.0001).

Administration of *B. thuringiensis *without antibiotics was lethal to all six species, and antibiotics significantly reduced mortality in five of them (*M. sexta, V. cardui, P. rapae, L. dispar *and *H. virescens*) (Figure 1). Mean larval mortality of *V. cardui*, *M. sexta*, *P. rapae*, and *H. virsescens *reared without antibiotics and fed *B. thuringiensis *cells, spores, and toxin (DiPel) ranged from 63 to 100%. The same concentration of *B. thuringiensis *caused only 0 to 10% mortality in *V. cardui*, *M. sexta*, *P. rapae*, and *H. virsescens *when larvae were reared on diet amended with antibiotics. Similarly, mean mortality of *L. dispar *fed *B. thuringiensis *Cry1Ac toxin (MVPII) at 10 μg/ml of diet was reduced from 50% without antibiotics to 11% with antibiotics. *P. gossypiella *responded quite differently. Rearing on antibiotics significantly increased *P. gossypiella *mortality from 33% to 75% when fed 10 μg/ml of *B. thuringiensis *Cry1Ac toxin (MVPII).

Administration of antibiotics greatly delayed the time required for *B. thuringiensis *to kill *V. cardui, P. rapae, H. virescens*, and *L. dispar *larvae, and could not be calculated in *M. sexta *because there was no appreciable mortality (Table 2). Antibiotic feeding increased LT_50 _values in the range of 10-fold in *H. virescens*, 15-fold in *V. cardui *and 4-fold for *L. dispar *as compared with control larvae. *P. gossypiella *LT_50 _values were not affected by rearing on antibiotics or *B. thuringiensis *treatment.

**Table 2:** Effect of antibiotics on rate of mortality by *B. thuringiensis *(Bt) and *Enterobacter *sp. NAB3 (EntB) on six Lepidoptera species.

		**LT_25 _(95% FL) ^a^**		**LT_50 _(95% FL)**	
**Species**	**Bacterial treatment**	**No antibiotics**	**Antibiotics**	**No antibiotics**	**Antibiotics**

*Vanessa cardui*	untreated	no toxicity	no toxicity	no toxicity	no toxicity
	*Enterobacter*	no toxicity	NA^b^	no toxicity	NA
	Bt DiPel 25 IU	1.80 (1.54–2.26)	13.20 (7.83 – >100)*	1.91 (0.83–2.25)	29.48 (12.44 – >100)*
	Bt DiPel 25 IU + EntB	NA	1.62 (1.24–1.95)#	NA	2.61 (2.21–2.97)#

*Manduca sexta*	untreated	no toxicity	no toxicity	no toxicity	no toxicity
	*Enterobacter*	no toxicity	NA	no toxicity	NA
	Bt DiPel 25 IU	1.89 (1.48–2.23)	no toxicity	3.04 (2.63–3.44)	no toxicity
	Bt DiPel 25 IU + EntB	NA	3.81 (3.24–4.31)	NA	5.95 (5.23–7.16)

*Pieris rapae*	untreated	10.34 (7.36–806.32)	no toxicity	16.62 (9.67–>100)	No toxicity
	*Enterobacter*	6.91 (5.21–14.02)	no toxicity	16.57 (9.79–96.47)	no toxicity
	Bt DiPel 25 IU	1.26 (1.02–1.47)	12.95 (7.83–>100)*	1.76 (1.52–1.99)	27.38 (12.04–>100)*
	Bt DiPel 25 IU + EntB	1.98 (1.66–2.27)#	1.47 (1.23–1.67)#	2.84 (2.52–3.16)#	1.98 (1.75–2.20)#

*Heliothis virescens*	untreated	no toxicity	NA	no toxicity	NA
	*Enterobacter*	no toxicity	NA	no toxicity	NA
	Bt DiPel 100 IU	0.48 (0.17–0.80)	8.62 (7.00–48.23)*	1.13 (0.80–1.77)	11.82 (8.48–267)*
	Bt DiPel 100 IU + EntB	NA	no toxicity	NA	no toxicity

*Pectinophora gossypiella*	untreated	no toxicity	no toxicity	no toxicity	no toxicity
	*Enterobacter*	no toxicity	no toxicity	no toxicity	no toxicity
	Bt MVPII 10 μg	17.87 (15.48–20.22)	15.46 (13.44–16.68)	20.99 (18.94–29.59)	17.56 (16.21–19.20)
	Bt MVPII 10 μg + EntB	7.11 (3.61–9.44)#	6.30 (2.78–8.62)#	13.71 (10.52–20.13)	12.61 (9.42–18.18)

*Lymantria dispar*	untreated	15.16 (8.90–398.16)	no toxicity	40.09 (16.05–>100)	no toxicity
	*Enterobacter*	no toxicity	no toxicity	no toxicity	no toxicity
	Bt MVPII 10 μg	4.97 (4.28–5.68)	9.52 (7.99–34.42)*	8.17 (6.95–9.41)	12.41 (10.40–125.70)*
	Bt MVPII 10 μg + EntB	NA	5.23 (4.62–5.81)#	NA	7.62 (6.76–9.24)#

Larval mortality rates were analyzed by PROC PROBIT. Estimates of the time (day) at which 25% and 50% of larvae died for each treatment are listed. Treatments with no significant mortality (*X*^2 ^> 0.05) are defined as having no toxicity. A cut-off of > 100 was assigned to upper FL estimates (note: these are computational outputs, not actual estimates of larval lifespan). Bt mortality rates denoted with * are significantly different (non-overlapping fiducial limits (FL)) with their respective antibiotic treatment. Values denoted with # are significantly different with the addition of *Enterobacter *sp. NAB3, as compared with Bt alone. ^a ^Units are days, ^b ^NA = Not tested

### Restoration of susceptibility to *B. thuringiensis *by Enterobacter sp. NAB3

Feeding *Enterobacter *sp. NAB3, a normal gut bacterium of *L. dispar*, restored *B. thuringiensis *killing ability to antibiotic-reared larvae of *M. sexta *(from 0% to 61%)*, V. cardui *(from 8% to 86%), *P. rapae *(from 8% to 100%) and *L. dispar *(from 11% to 44%) (Figure 1). Mortality following ingestion of *Enterobacter *sp. NAB3, by *V. cardui*, *M. sexta*, *H. virescens*, *P. gossypiella*, and *L. dispar *that had been reared on antibiotics did not differ significantly from untreated controls. However, larval mortality of antibiotic-treated *P. rapae *larvae following ingestion of *Enterobacter *sp. NAB3 was significantly higher than untreated controls (25% vs. 8%). In *P. rapae*, mortality was highest in larvae reared on antibiotics and fed the combination of *Enterobacter *sp. NAB3 and *B. thuringiensis*, though this mortality was statistically equivalent to that of larvae reared without antibiotics and fed *B. thuringiensis *(Figure 1).

In contrast to the effect of *Enterobacter *sp. NAB3 on *V. cardui*, *M. sexta*, *P. rapae *and *L. dispar*, the addition of the enteric bacterium *Enterobacter *sp. NAB3 from *L. dispar *did not restore *B. thuringiensis*-induced killing of *H. virescens *(3% mortality), even though rearing on antibiotics reduced susceptibility to *B. thuringiensis *(89% vs. 11%). In the case of *P. gossypiella*, though larval mortality to *B. thuringiensis *Cry1Ac was higher when larvae were reared on antibiotics, *Enterobacter sp*. NAB3 did not increase or decrease total mortality from *B. thuringiensis *toxin.

The impact of *B. thuringiensis *on time to death (LT_25 _or LT_50_) of *V. cardui*, *P. rapae *and *L. dispar *did not differ significantly between larvae reared on unamended diet and those fed *Enterobacter *sp. NAB3 following antibiotic-rearing (Table 2). In the case of *M. sexta*, total larval mortality was significantly lower and time to death greater in antibiotic-reared larvae fed *Enterobacter *sp. NAB3 than in larvae fed *B. thuringiensis *and reared on unamended diet (Figure 1, Table 2). There was no significant effect of antibiotics on the time to death (LT_50_) induced by *Enterobacter *sp. NAB3 on *P. gossypiella*. However, there was a significant effect of *Enterobacter *sp. NAB3 on *P. gossypiella *mortality due to *B. thuringiensis *in the first 12 days of the 21-day assay (Table 2). Feeding *Enterobacter *sp. NAB3 significantly reduced time to 25% mortality in *B. thuringiensis*-treated larvae, when they were reared on diet both without antibiotics (7.1 vs. 17.9 days) and amended with antibiotics (6.3 vs.15.5 days).

## Discussion

These results indicate that enteric bacteria have important roles in *B. thuringiensis*-induced killing of Lepidoptera across a range of taxonomy, feeding breadth, and relative susceptibility to *B. thuringiensis*. This impact of enteric bacteria differs among species. Oral administration of antibiotics reduced populations of gut bacteria of all five species in which they were initially detectable, and likewise reduced larval mortality due to *B. thuringiensis *of five of the six species tested. Feeding *Enterobacter *sp. NAB3 to antibiotic-reared larvae restored susceptibility to *B. thuringiensis *in four of these five species. These four Lepidoptera contained gut bacteria closely related to *Enterobacter s*p. NAB3, which might serve a similar role in larval susceptibility to *B. thuringiensis*.

The similar results with the cell-free formulation of *B. thuringiensis *toxin Cry1Ac (MVPII), *E. coli *producing Cry1Aa, and the formulation of cells, spores, and toxin (DiPel) in *L. dispar*, that is, the reduction of susceptibility by antibiotics and the restoration of mortality by *Enterobacter *sp. NAB3, indicate that our previous results were not specific to a particular *B. thuringiensis *formulation. In addition, this result substantiates our previous evidence [27,37] that the effect of antibiotics on *B. thuringiensis *susceptibility is not due to direct effects on the *B. thuringiensis *bacterium, as killing was reduced with the *B. thuringiensis *cell-free formulation containing only encapsulated Cry1Ac toxin.

The inability of *Enterobacter s*p. NAB3 to restore *B. thuringiensis*-induced killing of *H. virescens *indicates that while antibiotics may alter larval susceptibility to *B. thuringiensis*, the mechanism by which gut bacteria mediate *B. thuringiensis*-induced killing requires further elucidation, as do the different responses to gut bacteria in various insect species. We were unable to detect gut bacteria in *H. virescens*, which deserves further study to determine whether it has an as-yet-undetected gut microbiota. Sampling of additional populations, including field-collected larvae and additional methods to detect microorganisms of non-bacterial origin are needed to assess their role in *B. thuringiensis*-induced killing.

Interestingly, *P. gossypiella*, the only species in which antibiotic treatment did not reduce, and actually increased, susceptibility to *B. thuringiensis*, was also the only species that was singularly associated with a Gram-positive bacterium, *Enterococcus casseliflavus*. This suggests that *E. casseliflavus *might protect its host from killing by *B. thuringiensis*. Such a protective role has been proposed for gut bacteria of the tortricid *Homona magnanima *(Diakonoff) [38], in which Firmicutes related to *E. casseliflavus (Staphylococcus *and *Streptococcus *spp.) reduce *B. thuringiensis *growth in cadavers. We previously reported that a related bacterium (*Enterococcus faecalis*) was no longer detectable in the guts of *L. dispar *larvae reared on antibiotics, but unlike *Enterobacter *sp. NAB3, this bacterium did not restore larval susceptibility to *B. thuringiensis *[27]. It is also noteworthy that *P. gossypiella *is much more closely related to *H. magnanima *than to the other five species tested in the present study . The lack of Gram-negative gut bacteria (Proteobacteria), coupled with findings reported by others, suggests that the mechanism of *B. thuringiensis*-induced killing differs between *P. gossypiella *and other lepidopteran species [39,40]. While *Enterobacter *sp. NAB3 did not increase final mortality of *P. gossypiella *due to *B. thuringiensis*, it reduced the time until death, with or without antibiotics. This effect is noteworthy given that larvae were exposed to *Enterobacter *sp. NAB3 for only the first two days of the assay, while they were exposed to *B. thuringiensis *for the remaining 21 days, because obtaining treatment effects required much longer exposure to *B. thuringiensis *in *P. gossypiella *than in the other Lepidoptera used in this study.

Though general descriptions have been proposed to categorize host responses to *B. thuringiensis *[14], studies have demonstrated that *B. thuringiensis *susceptibility is influenced by diverse factors including the insect host [12,37,41-44], *B. thuringiensis *strain [45-47], and environmental conditions [48,49]. Our results demonstrate that in addition to these previously described factors, larval enteric bacteria affect susceptibility to *B. thuringiensis*, and the extent of this impact varies across lepidopteran species. These factors are not mutually exclusive and in some cases may interact, as, for example, host diet can alter the composition of enteric bacteria [50,51]. From a pest management perspective, the ability of a non-specific enteric bacterium from *L. dispar *to restore *B. thuringiensis*-induced mortality of other lepidopteran species may provide opportunities for increasing susceptibility or preventing resistance. Moreover, this contribution of enteric bacteria to host mortality suggests that toxin feeding causes a transition of otherwise benign bacteria into opportunistic pathogens in some, but not all hosts. These associations between *B. thuringiensis *toxin and the gut microbiota of Lepidoptera may provide a useful model with which to identify the factors involved in the induction of adverse effects by normally beneficial or benign bacteria.

## Conclusion

We tested the role of gut bacteria in larval susceptibility to *B. thuringiensis *among six species of Lepidoptera representing six families. Gut bacteria are required for *B. thuringiensis*-induced mortality of four of these, *Manduca sexta*, *Pieris rapae*, *Vanessa cardui*, and *Lymantria dispar*. This work also demonstrates that gut bacteria are not required for *B. thuringiensis*-induced killing of all Lepidoptera. A reduction of gut bacteria increased *P. gossypiella *susceptibility to *B. thuringiensis*, and antibiotic treatment reduced *B. thuringiensis*-induced mortality of *Heliothis virescens *larvae, even though no bacteria tested restored killing. *H. virescens *presents an intriguing model in which bacteria may play a role, but in a more complex manner than in the other host species.

## Methods

### Insect selection and rearing

The six insect species were selected primarily for their representation of a range of families across Lepidoptera. Additionally, these species represent a range of feeding breadths from polyphagous to monophagous and are exposed to a broad array of phytochemicals, factors that are known to influence *B. thuringiensis *susceptibility. They are also economically and ecologically important as either agricultural pests or valued biodiversity indicators.

Eggs of *M. sexta*, *V. cardui*, and *P. rapae *were obtained from Carolina Biological Sciences (Burlington, NC, USA). Eggs of *H. virescens *strain YDK were provided from laboratory colonies of F. Gould (North Carolina State University, Raleigh, NC, USA) or purchased from Benzon Research (Carlisle, PA). *P. gossypiella *strain AF28 was provided by B. Tabashnik (University of Arizona, Tuscon, AZ, USA). Eggs of *L. dispar *were obtained from culture NJSS at USDA-APHIS (Cape Cod, MA). All eggs were surface sterilized with a solution of Tween-80 (polyoxyethylene sorbitan monooleate), bleach, and distilled water as described previously [52]. Larvae of all species were reared in 15 mm Petri dishes on sterilized artificial diet (USDA, Hamden Formula: *M. sexta*, *V. cardui*, *P. rapae*, *H. virescens*; amended USDA, PBW formula: *P. gossypiella*) or sterilized artificial diet amended with antibiotics (500 mg/l of diet each penicillin, gentamicin, rifampicin, streptomycin). Larvae were reared in an environmental chamber with a 16:8 (L:D) photoperiod at 25°C.

### Bacterial and toxin strains

The *B. thuringiensis *used in assays with *M. sexta*, *V. cardui*, *P. rapae*, and *H. virescens *was a commercial formulation of *B. thuringiensis *subsp. *kurstaki *(DiPel^® ^TP, Valent Biosciences, Libertyville, IL, USA), consisting of cells, toxins (Cry1Aa, Cry1Ab, Cry1Ac, and Cry2A), and spores. Assays conducted with *P. gossypiella *and *L. dispar *used the MVP ™II formulation of *B. thuringiensis *toxin (Cry1Ac encapsulated in *Pseudomonas fluorescens*, Dow AgroSciences, San Diego, CA USA). *Enterobacter *sp. NAB3 was originally isolated from the midguts of gypsy moth larva feeding on sterile artificial diet [50]. For feeding assays, *Enterobacter *sp. NAB3 was cultured by shaking overnight in half-strength tryptic soy broth at 28°C. The overnight culture was washed once and resuspended in 1× phosphate-buffered saline (PBS) pH 8.0 prior to use in assays.

### Mortality assays

Assays for *M. sexta, V. cardui, P. rapae*, and *H. virescens *were performed as previously described for *L. dispar *[27]. All treatments were applied to sterile artificial diet without antibiotics. Assays were performed with third-instar larvae of *M. sexta, V. cardui*, and *H. virescens*. In the case of *P. rapae*, assays were performed using fourth-instar larvae. A concentration of 25IU, or for *H. virescens *100IU, was surface applied in a volume of 1 μl to a standard diet disk (3 mm diameter, 1 mm height) and fed to the larvae on two consecutive days. In the case of *P. gossypiella*, a standard protocol [39] using Cry1Ac diet incorporation with the diagnostic concentration of 10 μg/ml of diet was employed. *P. gossypiella *larvae were provided diet with *B. thuringiensis *Cry1Ac for the duration of the assay. To provide comparison with previous results, the MVP™II formulation was also tested with *L. dispar *at 10 μg/ml of diet with the surface application method described above. For establishment of *Enterobacter *sp. NAB3, larvae reared on antibiotics were fed 1 μl of a washed overnight culture (approximately 10^6 ^cells) for two days. For *P. gossypiella, Enterobacter *sp. NAB3 was surface applied to diet containing *B. thuringiensis *Cry1Ac for two days, after which larvae were transferred to fresh diet containing *B. thuringiensis *Cry1Ac. Mortality was recorded every 24 hours for 7 days. In the case of *P. gossypiella*, mortality recordings were extended to 21 days according to established protocols [39].

### Analysis of midgut bacteria 16S rRNA genes

Larvae were surface sterilized for 5s in 95% ethanol prior to dissection. The crop and midgut of 10 larvae of each species were pooled and total microbial DNA was extracted as described previously [50]. Bacterial 16S rRNA genes were amplified by PCR from total DNA using primers 27F-HT and 1492R-HT [53]. Clone libraries were constructed with the pGEM-T Vector system (Promega, Madison, WI) and electrocompetent *E. coli *JM109 cells. Purified plasmid DNA was amplified using the plasmid primers M13F and M13R and sequenced with 27F-HT. All 16S rRNA gene sequences (~700 bp) were compiled using the SeqMan program from the DNAStar software package (DNASTAR, Inc., Madison, WI) and compared with available databases with BLAST to determine phylogenetic affiliations.

### Statistics

Mean larval mortality and standard error were determined from three replications of 12 larvae each using PROC MEANS [54]. Means were separated using Fisher's LSD at *P *= 0.05. The effect of *Enterobacter *sp. NAB3 on time to death of *B. thuringiensis *treated larvae was analyzed using PROC PROBIT [54]. Significant differences in LT_25 _and LT_50 _values between treatments were determined based on probit values with non-overlapping 95% Fiducial Limits [36].

## Authors' contributions

NAB, MDM, and JHo reared test larvae and performed larval bioassays. NAB, MDM, and CJR carried out the molecular analysis of gut bacteria. NAB performed the statistical analysis of the data. NAB, JHa, and KFR conceived of and designed the study and MDM participated in its design. NAB, JHa and KFR analyzed the data and wrote the manuscript. All authors read and approved the final manuscript.
